# New Strategies and In Vivo Monitoring Methods for Stem Cell-Based Anticancer Therapies

**DOI:** 10.1155/2018/7315218

**Published:** 2018-11-15

**Authors:** Ping Wang, Aitor Aguirre

**Affiliations:** ^1^Precision Health Program, Department of Radiology, College of Human Medicine, Michigan State University, East Lansing, MI 48823, USA; ^2^Division of Developmental and Stem Cell Biology, Institute for Quantitative Health Science and Engineering, Michigan State University, East Lansing, MI 48823, USA; ^3^Department of Biomedical Engineering, College of Engineering, Michigan State University, East Lansing, MI 48823, USA

## Abstract

Cancer is a devastating disease and the second cause of death in the developed world. Despite significant advances in recent years, such as the introduction of targeted therapies such as receptor tyrosine kinase inhibitors and immunotherapy, current approaches are insufficient to stop the advance of the disease and many cancer types remain largely intractable. In this review, we describe the latest and most revolutionary stem cell-based approaches for the treatment of cancer. We also summarize the emerging imaging modalities being applied for monitoring anticancer stem cell therapy success and discuss the implications of these novel technologies for precision medicine.

## 1. Introduction

**Figure 1:**
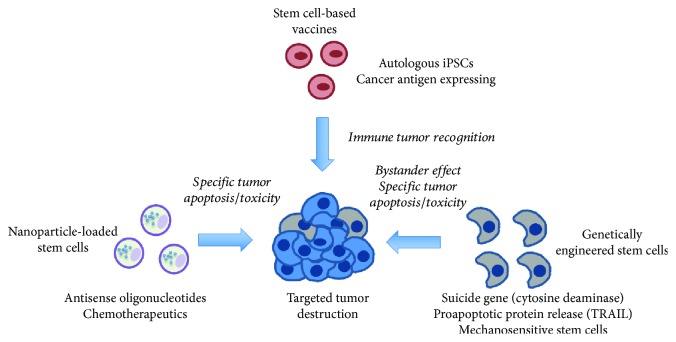
Stem cell-based strategies for anticancer therapy. Tumors can be specifically targeted with stem cells to make them vulnerable to therapy. Top: stem cell-based vaccines leverage the similarities between cancer cells and stem cells to promote immune tumor recognition; left: nanoparticle-loaded stem cells exhibit efficient homing to tumors, where they deliver their payload in the form of chemotherapies or apoptosis-inducing oligonucleotides; right: genetically engineered stem cells can express and release proapoptotic proteins or ligands in the tumor microenvironment or contain enzymes metabolizing prodrugs to their cytotoxic form (e.g., cytosine deaminase). Stem cells can also be engineered to recognize biophysical features of the tumor microenvironment before activating their engineered cytotoxic program.

Cancer is the second cause of death of men and women in the United States and a major health problem worldwide [1]. Mortality data for 2018 predicts 1.7 million new cancer cases and 0.6 million cancer-related deaths only in the US [2]. There is however room for hope. Among the top 10 causes of death, cancer is the only one steadily declining (about 26% for men and women in the US in the last 25 years), reflecting continuous improvements in diagnosis, care, and treatment [2]. Therapeutic intervention has significantly advanced in the last two decades, particularly with the introduction of targeted therapeutics such as receptor tyrosine kinase inhibitors (e.g., erlotinib in 2003) [3, 4] and immunotherapy (e.g., pembrolizumab in 2014) [5, 6]. These compounds exhibit much higher selectivity for cancer cells over conventional treatments and minimize side effects. Unfortunately, despite the extensive efforts invested in clinical development of cancer therapeutics, many cancers remain difficult or impossible to treat by traditional approaches. Furthermore, tumors evolve under treatment, and cells become widely chemoresistant and highly invasive, reducing treatment options as the disease progresses [7, 8]. An innovative approach for cancer treatment in recent years is the use of stem cell-based therapies [9, 10]. In this context, rather than regenerating, repairing, or replenishing tissues, stem cells are carriers that infiltrate tumors to deliver lethal payloads and tell us about the mechanisms of cancer cell survival and immune evasion. Stem cells possess at least two unique biological characteristics that make them ideally suited to fight cancer. For starters, embryos and tumors share many characteristics, including surface antigens, production of growth factors, and the capacity to evade, at least partially, the immune system [9]. In 1838, these similarities led Muller to formulate what could be considered the first stem cell theory of cancer origin (still highly controversial) [11]. In 1906 Schone would show that vaccination of animals with fetal tissues could render them partially resistant to cancer, demonstrating the close connection existing between cancer cells and stem cells [11]. More recent efforts have established beyond any doubt that stem cells and cancer cells share many common features at the molecular level, including the activation of developmental signaling pathways promoting cell survival, proliferation, self-renewal, and tissue invasion (e.g., Wnt, Notch, Hippo, and epithelial to mesenchymal transition) [12, 13]. It might be due to these similarities that stem cells also exhibit strong tropism towards tumors, which in turn makes them attractive candidates for targeted delivery of drugs or other compounds with minimal side effects. Strategies for fighting cancer with stem cell-based therapies fall into two broad categories: (1) stem cell vaccines, using the identity property, and (2) stem cell carriers, exploiting their tumor-tropic behavior. The different strategies and examples of their use can be found in Figure 1. Additionally, Table 1 includes a number of ongoing clinical trials in the US using stem cell-based therapies for anticancer treatment to highlight the relevance of this growing field for translational applications.

## 2. Stem Cell-Based Antitumor Vaccines

The idea of generating immunity against cancer cells (immunotherapy) is not new and has been pursued for many decades. In the 19th century, scientists noticed the similarity between embryonic cells and cancer cells. It was observed that when mice had been exposed to fetal tissue from another mouse, the recipient would reject transplanted tumors (for a detailed review on this topic, see [11]). These ideas were explored in-depth during the following decades, particularly during the 60s and 70s. Stem cells and cancer cells share significant cellular and molecular properties. Immunization with embryonal material was enough to prevent tumor growth and to suppress tumor formation by administration of carcinogens [11]. However, due to technical and ethical limitations at the time (inoculation of human fetal tissue would not be feasible in humans), these approaches were progressively abandoned. This changed at the turn of the century, with the enormous expansion in the stem cell biology in the last two decades, the establishment of numerous human embryonic stem cell lines and the introduction of induced pluripotent stem cells [14, 15]. These advances render the previous ethical and social concerns associated with fetal immunization obsolete, leading to a resurging interest in human anticancer stem cell-based vaccines.

In a breakthrough study published very recently, Kooreman et al. reported using irradiated induced pluripotent stem cells in conjunction with adjuvant therapy to vaccinate mice against a wide number of cancer types, including breast cancer, mesothelioma, and melanoma with great success [16]. Using RNA-seq, the authors found that iPSCs and cancer cells possessed a similar signature in several potential cancer antigens, suggesting that iPSCs could be used to prime the host's immune system. This approach has several advantages, including use of autologous cells minimizing host rejection and exposure to known and unknown cancer-associated antigens simultaneously to promote a more solid immune response against the tumor. As a proof of concept, the authors injected mouse iPSCs in mice and then transplanted cancer cells (breast, melanoma) in subcutaneous and orthotopic models. In all cases, a spectacular regression of the tumors was observed when compared with nonvaccinated control mice [16]. It was possible to determine that B and T lymphocytes were primarily responsible for this activity. Although iPSC vaccination was successful in preventing or reducing tumor growth, it was insufficient as a therapy to prevent the growth of established tumors, suggesting tumor immunosuppressive mechanisms might be still too strong [16]. Combination of iPSC-based vaccines with targeted immunotherapy might be an interesting avenue for treatment in the future. Of note, the authors did not detect any negative effects derived from iPSC vaccination, such as autoimmunity or teratoma formation, making this approach more attractive for translation into the clinic.

An alternative approach at stem cell-based anticancer vaccination is the use of iPSCs to derive dendritic cells (DCs), which play an essential role in T cell activation, engineered to express tumor-specific antigens. Kitadani et al. applied this concept to the treatment of gastrointestinal cancer [17]. After generating iPSC-derived DCs (iPSDCs) with typical DC markers and cytokine secretion, iPSDCs were engineered to express carcinoembryonic antigen (CEA) employing adenoviral transduction. T cells from healthy human donors were exposed to the CEA-iPSDCs in vitro and then c-cultured with a panel of gastrointestinal cancer cell lines. Human T cells responded with strong cytotoxic activity to cancer cells expressing CEA, but not others [17]. Transplantation of mouse iPSDCs into a mouse subcutaneous model of gastrointestinal cancer resulted in remarkable cytotoxicity and tumor growth inhibition (~4-fold smaller tumor volume compared to controls) [17]. More studies will be necessary to determine the safety and efficacy of stem cell-based vaccines for controlling or eradicating human tumors; however, present advances support their feasibility and guarantee further research.

## 3. Targeted Suicide Stem Cells to Destroy Tumors

Stem cells have a significant capacity to home to tumors due to shared chemotactic and signaling pathways with cancer cells [9]. This property can be exploited for targeted therapies by placing specific stem cell types into the tumor mass. Protocols for growing and maintaining many different types of tissue-specific stem cells are now available and have significantly improved over the last decade [18, 19]. Introducing genetic modifications in stem cells is now easier than ever before since the implementation of CRISPR technologies [20]. These technical advances facilitate the making of engineered stem cell types for targeting tumors based on their origin and characteristics with minimal side effects in other tissues or organs. Successful attempts following this strategy can be found for the treatment of aggressive brain tumors, which are among the most deadly and challenging cancers to treat. Survival for patients with glioblastoma multiforme (GBM) ranges from 12 to 15 months, and treatment options are very limited and consist on aggressive chemo and radiotherapy [21]. Attempts to efficiently target glioblastoma have traditionally been insufficient for sustained therapeutic benefit [21]. Using a transdifferentiation approach, Bagó et al. generated autologous neural stem cells from skin fibroblasts for theranostic applications (iNSCs) [22]. The authors determined that iNSCs exhibited strong tumor-homing activity towards GBM cell lines due to CXCR4 chemotaxis [22]. The authors decided to genetically engineer iNSCs to express a secreted variant of the proapoptotic molecule TRAIL (TNF-related apoptosis-inducing ligand) and transplanted cells intravenously into mice carrying human glioblastoma xenografts. Over a period of 3 weeks, iNSCs lead to a 250-fold tumor mass reduction and a median increase in survival from 22 to 49 days. Other genetic modifications of iNSCs, such as ganciclovir prodrug therapy, were also successful. To mimic the postoperative setting in humans, mice with xenografted GBM were subjected to surgical resection and iNSC therapy. The treatment delayed the regrowth of GBMs 3-fold and extended survival from 46 to 60 days [22]. Taken together, these results suggest that tumor-homing stem cells are a powerful option for therapy, particularly in inaccessible tumors. Locally residing stem cells are present in almost every tissue in the body, potentially making this approach applicable to other tumor types.

## 4. Targeting the Metastatic Niche with Mechanosensitive Stem Cells

Another aspect of cancer that can be tackled with stem cell therapy is metastasis. Cancer metastases are responsible for 90% of cancer deaths [23, 24]. Treatments to directly target metastatic tumors are sorely lacking, and surgical resection is not always a feasible option, particularly when multiple metastatic sites are present. Interestingly, metastases frequently differ from their tissues of origin by possessing largely altered extracellular microenvironments, which leads to altered matrix stiffness, particularly in tumors with strong lysyl oxidase (LOX) expression (e.g., breast cancer metastases have 15-fold increased stiffness versus normal breast tissue) [25, 26]. Taking these properties into consideration, Liu et al. hypothesized that a mechanosensitive mesenchymal stem cell-based system, named mechanoresponsive cell system (MRCS), could be created to target cancer metastases [27]. Systemically infused MSCs target tumor sites due to naturally occurring combinations of tumor tropic molecules (growth factors, cytokines), and matrix stiffness is an important contributor to their behavior, including chemotaxis and differentiation, indicating that a fine-tuned mechanoresponsive machinery is already present in this cell type [27, 28]. Autologous MSCs can be easily obtained from a patient's adipose tissue or differentiated from iPSCs and expanded *in vitro* [29]. MSCs sense and transduce extracellular mechanical cues through the Hippo pathway effector YAP. In soft substrates, YAP remains in the cytoplasm in its inactive form, while hard substrates promote YAP nuclear translocation and associated transcriptional programs [30]. Taking advantage of this property, the authors genetically engineered MSCs to express the suicide gene cytosine deaminase (CD) under the control of the YAP promoter (referred to as CD-MCRS) [27]. In this system conditions, systemically infused CD-MCRS cells are attracted to metastatic sites and, once exposed to the matrix stiffness present at those locations, start expressing CD. Administration of 5-fluorocytosine at this point specifically kills metastatic cells by the bystander effect. To provide proof of concept on this approach, Liu et al. used mice transplanted with MDA-MB-231 breast cancer cells into the lung to mimic metastatic spread [27]. As expected, infused CD-MCRS homed to the lungs and activated CD expression. When administered in conjunction with 5-fluorocytosine, metastatic tumors shrank 2- to 3-fold and treated animals experienced significantly improved survival [27]. Overall, these results show great promise for the treatment of metastatic cancer and highlight the importance of using the biophysical properties of the tumor environment for targeted therapies.

## 5. Stem Cells as a Nanoparticle Delivery System

The tumor homing characteristics of stem cells can be leveraged to specifically deliver particles to tumor sites [31]. In a similar approach to the one described before for metastatic mechanosensing, MSCs can be induced to take up drug-loaded nanoparticles. Zhao et al. attempted this approach by loading MSCs with doxorubicin-containing poly-lactic-coglycolic acid nanoparticles (PLGA-DOX) [32]. MSCs readily took up PLGA-DOX and, due to low bioavailability of the drug in this composition, received low cytotoxicity. When transplanted into mice bearing lung metastases, MSCs homed to the tumor sites and locally released DOX, resulting in a significantly reduced number of tumor nodules (~3-fold reduction) [32]. An interesting recent variation of this approach involves the use of a physical phenomenon known as magnetic hyperthermia. Magnetic hyperthermia consists on the heating of tissues by means of magnetic nanoparticles and alternating magnetic fields (AMF). MSCs can be loaded with superparamagnetic iron oxide nanoparticles (SPIONs), which have minimal toxicity [33]. Once MSCs have homed into the tumor, SPIONs are delivered to the surrounding cells by exosome delivery. At this point, application of a high-frequency AMF will produce a localized hyperthermic effect reaching temperatures of 42–45 Celsius at intervals of 20 minutes. This localized effect exerts extensive damage to tumor cells in vitro, with up to 80% growth inhibition [33]. Although very promising, stem cell-mediated-magnetic hyperthermia therapeutic effects have not been explored *in vivo* in enough detail yet.

## 6. Stem Cells as Oncolytic Virus Carriers

Oncolytic viruses are an emerging class of cancer therapeutics. In 2017, the FDA approved the first immunogenic oncolytic virus (OV) for therapy in advanced melanoma [34]. However, the systemic administration of OV can lead to serious side effects. To circumvent this problem, Du et al. designed a strategy in which MSCs could act as oncolytic herpes simplex virus (oHSV) carriers into the tumor mass [34]. Using a mouse model that closely recapitulates melanoma progression, the authors demonstrated the therapeutic efficacy of oHSV-loaded MSCs. The cells, which were delivered via carotid artery, efficiently homed to the tumor metastases and reduced tumor size and foci number by ~2- to 4-fold [34]. Furthermore, treatment also extended survival by approximately 20 days. The authors have also demonstrated the efficacy of this approach in other tumor types before [35]. Overall, oncolytic virus therapies remain a strong option for therapy in the close future and this study demonstrates that further refinement using stem cells as carriers can improve therapeutic outcomes and minimize side effects.

## 7. Stem Cell Reprogramming Technologies for Cancer Immunotherapy

The field of cancer immunotherapy has seen important advances in recent years, including several clinical trials and approval of pembrolizumab. Transfer of T cell receptor (TCR) genes into patients' peripheral T cells has achieved good clinical outcomes [36], indicating that targeting a single antigen can be effective for some types of cancer. In addition, T cell expressing chimeric antigen receptor (CAR), an engineered receptor molecule, which combines an antibody recognition domain and cytoplasmic signaling domains, has demonstrated therapeutic effect in a certain type of leukemia [37]. In both TCR and CAR engineering, peripheral T cells are transduced by a retrovirus, bringing about the risk of tumorigenicity due to the random integration of a transfected gene into the genome. Moreover, in the autologous setting, it would be costly to produce T cells. To address this issue, a strategy has been proposed to regenerate T cells utilizing iPSC technology. Themeli et al. reported that CAR-expressing T cells were regenerated from iPSCs transduced with a CAR gene. iPSCs were generated by retrovirus reprogramming T cells isolated from peripheral blood of healthy donors. The CAR sequence specific for CD19 was inserted into iPSCs using CRISPR/Cas9. iPSC-derived CAR specific-T cells were phenotypically similar to innate T*γδ* cells and showed ability to inhibit tumor growth in a xenograft animal model [38–40]. This strategy brings hope that CAR-T therapy may also work for other types of cancer beside hematologic malignancies. Certain obstacles associated with CAR-T including further customization of the technology to recognize specific/other tumor types and predicting and limiting cross-reactivity need to be resolved. CRISPR/Cas9 technologies will be useful to target CAR-T gene constructs to genomic safe harbor sites, reducing the risk of undesired effects. However, challenges related to the use of this cutting-edge technology for human therapy remain. Two recently published studies reported that stem cells whose genomes were successfully edited by CRISPR-Cas9 had the potential to be tumorigenic themselves due to p53 mutations. These results indicated that p53 and related genes should be monitored when developing stem cell-based therapies utilizing CRISPR-Cas9 [41, 42].

## 8. New Imaging Modalities for Stem Cell Theranostics in Cancer

**Figure 2:**
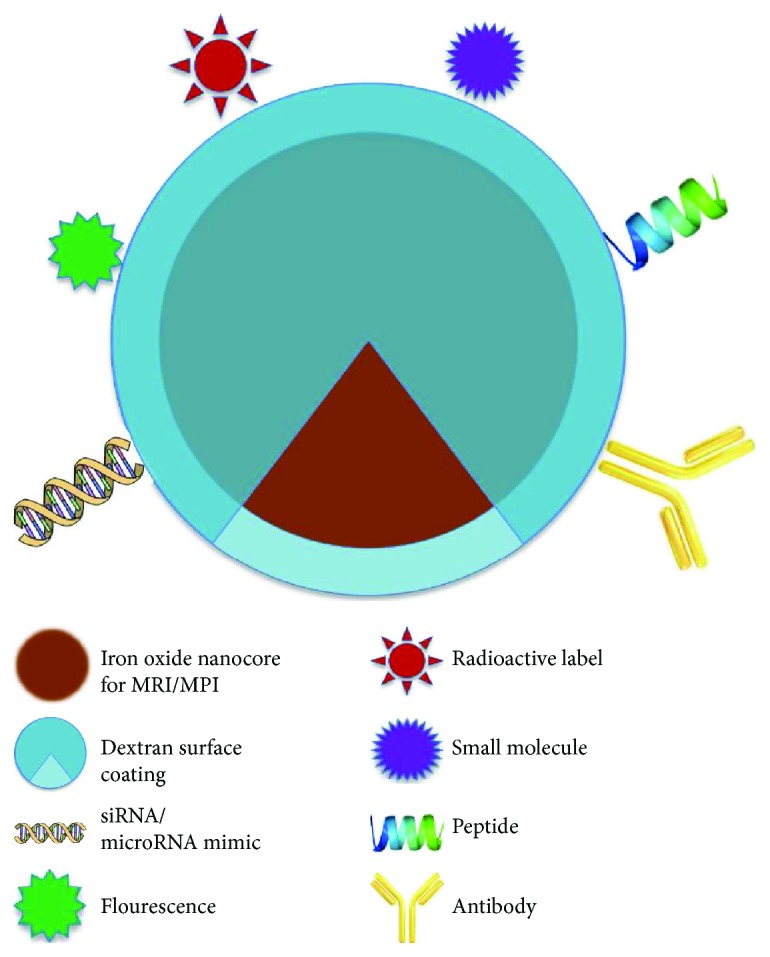
Theranostic magnetic nanoparticles for stem cell anticancer therapy. Iron oxide nanoparticles can be functionalized by applying a dextran coating. Different biologically active substances (antibodies, RNA/DNA, and drugs) intended to target or damage the tumor, or labeling probes for tracing and diagnostics, can be then tethered to the nanoparticle for theranostic applications.

Stem cell-based therapies have enormous potential for cancer treatment. For maximal efficacy, these promising therapies require targeted cell delivery to specific sites followed by successful cell engraftment. Various imaging methods have been applied for in vivo tracing of stem cells, including optimal imaging, nuclear imaging, and magnetic resonance imaging. Each imaging modality has its advantages and limitations in terms of sensitivity, tissue penetration, spatial resolution, and clinical potential [43, 44]. Optical imaging is a group of technologies that produce the image formed by the light rays from a self-luminous or an illuminated object that traverse an optical system. So far, this method is mainly restricted in tracking transplanted cells in animal models. In addition, low spatial resolution and limited tissue penetration are some disadvantages related to this method [45]. Nuclear imaging is based on the use of radiolabeled ligands targeting cell-specific antigens, receptors, metabolites, or pharmacologic agents. Many isotopes and labeling strategies have been investigated for stem cell labeling for nuclear imaging [46]. Lack of available isotopes, low spatial resolution, poor cellular uptake, and potential negative affect in cellular proliferation are some weaknesses of this modality. Magnetic resonance imaging (MRI) has some advantages over other modalities used for in vivo cellular imaging. It is clinically applicable and does not use radiation; it has high spatial resolution and unlimited tissue penetration, which can provide anatomical information of localizing transplanted cells. For MRI detection, stem cells usually need to be labeled with imaging contrast agents before transplantation. Superparamagnetic iron oxide (SPIO) nanoparticles, which result in signal-intensity voids or hypointense regions in T2-weighted or T2^∗^-weighted MR images [43], are the most common imaging probes used for tracking transplanted stem cells using MRI. However, signal voids in MR images produced by iron-labeled cells/cell clusters were difficult to distinguish from other low MR signals produced by tissue including intestine and blood vessel structures or artifacts [47]. Magnetic particle imaging (MPI) is an emerging imaging technique introduced in 2005 that directly identifies the intense magnetization of SPIOs rather than indirectly detecting SPIOs via signal dropouts, which could potentially overcome the disadvantages of cell tracking with MRI. MPI unambiguously detects superparamagnetic iron oxide nanoparticles with high specificity and sensitivity and other advantages over previous methods, such as the absence of background signal, linear quantitative ability, and high potential for clinic translation. MPI's great specificity results from its high image contrast, since magnetic particles serve as the only source for signal [48]. MPI's high sensitivity derives from the direct detection of the electronic magnetization of SPIO nanoparticles, which is 10^8^ times larger than the nuclear magnetization of protons seen in MRI [49], translating to a sensitivity to detect hundreds of magnetic nanoparticle-labeled cells with current hardware. MPI's safety is driven using clinically approved iron oxide nanoparticles, which have been proven safe for patients with compromised renal function. This new imaging modality has been tested for in vivo monitoring of transplanted stem cells. Zheng et al. firstly utilized MPI to monitor SPIO-labeled human embryonic stem cell- (hESC-) derived neural progenitor cells (NPCs) in vivo in a rat model. The results showed a 200-cell detection limit in vitro and in vivo, allowing them to monitor graft clearance over 87 days in the animal's brain using MPI [50]. In a more recent study, Zheng and colleagues imaged intravenously transplanted mesenchymal stem cells (MSCs) using MPI. Their studies demonstrated that labeled MSCs immediately entrapped in the lung tissue posttransplantation and then relocated to the liver within one day. Longitudinal MPI demonstrated a clearance half-life of MSC iron- oxide labels in the liver at 4.6 days [51]. These first in vivo MPI results indicate that MPI offers strong utility for quantitating transplanted stem cells labeled using SPIO. These results demonstrate that MPI's quantitative capacity arises from the linear signal change with nanoparticle concentration, which occurs independent of tissue depth. For clinical translation, a whole-body human MPI system for high-speed imaging is currently being developed and the first one has already delivered initial images. In terms of SPIO probe availability for MPI, there are several FDA-approved iron oxide nanoparticles under several brand names including ferucarbotran (Resovist®, Schering AG, Germany), ferumoxtran-10 (Sinerem®, Guerbet, France; Combidex® Advanced Magnetics Inc., MA, USA), and ferumoxytol (Feraheme®, AMAG Pharmaceuticals, Cambridge, MA) [52]. In addition, VivoTrax™ is provided for preclinical use by Magnetic Insight, CA, USA. Iron oxide nanoparticles used for cell labeling in MRI/MPI serve not only as imaging probes but also as nanocarriers for therapies. Various functional moieties could be attached to the coating of nanoparticle that serve as targeting macromolecules, therapeutic payloads, or additional imaging tags for multimodality in vivo imaging [53, 54]. These multifunctional nanodrugs could be carried by stem cells towards cancer cells for theranostic cancer treatment (Figure 2).

## 9. Perspectives and Conclusions

In conclusion, stem cell-based anticancer therapies offer great promise for the treatment of cancer. Although stem cells might be useful as cancer therapies of their own, they might also serve as powerful adjuvants in combination with traditional chemoradiotherapy treatment, or after surgery. Furthermore, cancer patient-derived iPSCs can be used to draw association between genotype and treatment responses and to identify biomarkers to inform patient selection for precision oncology [55, 56]. Emerging technologies such as CRISPR/Cas9 genome engineering and novel theranostic tools will be important factors in the successful implementation and continued improvement of stem cell-based anticancer therapies [57]. Many challenges remain, particularly regarding safety of stem cell transplants and CRISPR/Cas9 genomic manipulations. These issues are the focus of intense research and will be progressively clarified in the near future.

## Figures and Tables

**Table 1:** Current clinical trials using stem cell-based therapies for anticancer treatment in the US.

Title	NCT number	Status	Sponsors	Enrollment	Funding	Start	Location
Generation of Cancer Antigen-Specific T-cells From Human Induced Pluripotent Stem Cells (iPSC) for Research and Potential Future Therapy	NCT03407040	Enrolling by invitation	National Cancer Institute (NCI), National Institutes of Health Clinical Center (CC)	7000	NIH	30 Jan. 18	National Institutes of Health Clinical Center, Bethesda
Stem Cells in NF1 Patients with Tumors of the Central Nervous System	NCT03332030	Recruiting	Children's Research Institute	20	Other	27 Nov. 15	Children's National Medical Center, Washington
Neural Stem Cell Based Virotherapy of Newly Diagnosed Malignant Glioma	NCT03072134	Recruiting	Northwestern University	36	Other	24 Apr. 17	Northwestern Memorial Hospital, Chicago
Umbilical & Cord Blood (CB) Derived CAR-Engineered NK Cells for B Lymphoid Malignancies	NCT03056339	Recruiting	MD Anderson Cancer Center	36	Other	21 Jun. 17	University of Texas MD Anderson Cancer Center, Houston
Mesenchymal Stem Cells (MSC) for Ovarian Cancer	NCT02530047	Active, not recruiting	MD Anderson Cancer Center	5	Other	16 May 16	University of Texas MD Anderson Cancer Center, Houston
MV-NIS Infected Mesenchymal Stem Cells in Treating Patients with Recurrent Ovarian Cancer	NCT02068794	Recruiting	Mayo Clinic, National Cancer Institute (NCI)	54	Other, NIH	Mar. 14	Mayo Clinic, Rochester
Genetically Modified Neural Stem Cells, Flucytosine, and Leucovorin for Treating Patients with Recurrent High-Grade Gliomas	NCT02015819	Active, not recruiting	City of Hope Medical Center, National Cancer Institute (NCI)	18	Other, NIH	7 Oct. 14	City of Hope Medical Center, Duarte
Allogeneic Human Bone Marrow Derived Mesenchymal Stem Cells in Localized Prostate Cancer	NCT01983709	Terminated	Sidney Kimmel Comprehensive Cancer Center at Johns Hopkins	7	Other	Oct. 13	Johns Hopkins Hospital, Baltimore
Stem Cell Transplantation as Immunotherapy for Hematologic Malignancies	NCT00143559	Completed	St. Jude Children's Research Hospital	17	Other	Aug. 05	St. Jude Children's Research Hospital, Memphis

Data was obtained from NIH clinicaltrials.gov by performing searches for cancer-related clinical trials using the following terms: stem cell-based anticancer vaccine, engineered stem cells, targeted stem cell therapy, stem cell virus carrier, stem cell nanoparticle carrier, and stem cell immunotherapy.
